# Author Correction: Response of atomic spin-based sensors to magnetic and nonmagnetic perturbations

**DOI:** 10.1038/s41598-023-43890-5

**Published:** 2023-10-17

**Authors:** Mikhail Padniuk, Marek Kopciuch, Riccardo Cipolletti, Arne Wickenbrock, Dmitry Budker, Szymon Pustelny

**Affiliations:** 1 Marian Smoluchowski Institute of Physics, Jagiellonian University, Łojasiewicza 11, 30‑348 Kraków, Poland; 2 Helmholtz Institute, Johannes Gutenberg-Universitat at Mainz, 55099 Mainz, Germany; 3 Robert Bosch GmbH, Corporate Sector Research and Advance Engineering, Advanced Technologies and Micro Systems, 71272 Renningen, Germany

Correction to: *Scientific Reports* 10.1038/s41598-021-03609-w, published online 10 January 2022

The Acknowledgements section in the original version of this Article was incomplete, where the grant number of one of the funding agencies was omitted.

“SP would like to acknowledge support of the National Science Centre of Poland within the OPUS programme.”

now reads:

“SP would like to acknowledge support of the National Science Centre of Poland within the OPUS programme (2020/39/B/ST2/01524).”

The original Article has been corrected.

