# Supplementary material for: Response of atomic spin-based sensors to magnetic and nonmagnetic perturbations
Source: Sci Rep. 2022 Jan 10;12:324. doi: 10.1038/s41598-021-03609-w (PMC8748673; doi:10.1038/s41598-021-03609-w)
Supplement: Supplementary file 1 — Supplementary Information. [file 41598_2021_3609_MOESM1_ESM.pdf]

# Additional information

**Mikhail Padniuk[1,*], Marek Kopciuch[1], Riccardo Cipolletti[2,3], Arne Wickenbrock[2], Dmitry Budker[2], and Szymon Pustelny[1,+]**

[1]Marian Smoluchowski Institute of Physics, Jagiellonian University, Łojasiewicza 11, 30-348, Kraków, Poland.
[2]Helmholtz Institute, Johannes Gutenberg-Universitat at Mainz, 55099 Mainz, Germany
[3]Robert Bosch GmbH, Corporate Sector Research and Advance Engineering, Advanced Technologies and Micro Systems, 71272 Renningen, Germany
[*]michal.padniuk@doctoral.uj.edu.pl
[+]szymon.pustelny@uj.edu.pl

## ABSTRACT

Here, we provide supplementary materials for the article **Response of atomic spin-based sensors to magnetic and nonmagnetic perturbations**, which have been published in Scientific Reports.

## Nonmagnetic interactions in SERF and AM-NG co-magnetometers

### SERF magnetometer

The interaction-picture Hamiltonian, describing the evolution of AM electron spin $\mathbf{S}$ and AM nuclear spin $\mathbf{I}$ exposed to magnetic field $\mathbf{B}$ and generic nonmagnetic spin perturbation $\boldsymbol{\Xi}$, is given by

$$H = \mu_B g_s \mathbf{B}\cdot\mathbf{S} - \mu_B g_I \mathbf{B}\cdot\mathbf{I} + \chi_e \boldsymbol{\Xi}\cdot\mathbf{S} + \chi_I \boldsymbol{\Xi}\cdot\mathbf{I}, \tag{1}$$

where $\mu_B$ is the Bohr magneton, $g_S$ and $g_I$ are the electron and nuclear Landé factors, respectively, and $\chi_e$ and $\chi_I$ are the electron and nuclear constants associated with nonmagnetic coupling strength. Since $|g_I/g_S| \lesssim 10^{-2}$, we can simplify the Hamiltonian by neglecting the second term in Eq. (1).

In general, the anomalous field $\boldsymbol{\Xi}$ couples to protons and neutrons, thus we can factorise the nuclear coupling constant $\chi_I$ into neutron and proton contributions

$$\chi_I = \sigma_n \chi_n + \sigma_p \chi_p, \tag{2}$$

where $\sigma_n$ and $\sigma_p$ define the the neutron and proton fractional contributions to the total nuclear spin of the AM and $\chi_n$ and $\chi_p$ are the neutron and proton anomalous (dipole) coupling constants, respectively[1]. For the comparison of the response of the spins to the magnetic and anomalous couplings, it is convenient to redefine exotic spin couplings in terms of effective pseudo-magnetic field, which, for all perturbations yield

$$\mathbf{b_e} = \frac{\chi_e \boldsymbol{\Xi}}{g_s \mu_B}, \tag{3a}$$

$$\mathbf{b_n^{AM}} = \frac{\sigma_n \chi_n \boldsymbol{\Xi}}{g_s \mu_B}, \tag{3b}$$

$$\mathbf{b_p^{AM}} = \frac{\sigma_p \chi_p \boldsymbol{\Xi}}{g_s \mu_B}. \tag{3c}$$

Substituting the effective pseudo-magnetic field into Eq. (1) allows one to write the interaction Hamiltonian $H$ as

$$H = \mu_B g_S (\mathbf{B} + \mathbf{b_e})\cdot\mathbf{S} + \mu_B g_S (\mathbf{b_p^{AM}} + \mathbf{b_n^{AM}})\cdot\mathbf{I}. \tag{4}$$

Using the Liouville equation

$$\frac{d\rho}{dt} = \frac{1}{i\hbar}[H,\rho], \tag{5}$$

where $[,]$ denotes the commutator, to describe the evolution of the density matrix due to the Hamiltonian $H$ [Eq. (4)] allows us to calculate temporal evolution of the AM density matrix in the considered case

$$\frac{d\rho}{dt} = \frac{\mu_B g_S}{i\hbar}\left\{\left[(\mathbf{B} + \mathbf{b_e})\cdot\mathbf{S}, \rho\right] + \left[(\mathbf{b_n^{AM}} + \mathbf{b_p^{AM}})\cdot\mathbf{I}, \rho\right]\right\}. \tag{6}$$

At low fields and in the absence of hyperfine excitations, we can neglect hyperfine coherences in the AM atoms, which leads to a block-diagonal form of the AM density matrix

$$\rho = \begin{pmatrix} \rho_a & 0 \\ 0 & \rho_b \end{pmatrix}, \tag{7}$$

where $\rho_{a,b}$ corresponds to the $F_{a,b} = I \pm 1/2$ hyperfine state of the AM atoms[2]. The expectation values of AM electron spin $\langle \mathbf{S_{a,b}} \rangle$ and AM total atomic spin $\langle \mathbf{F_{a,b}} \rangle$ in the hyperfine states $a$ and $b$ are given by

$$\langle \mathbf{S_{a,b}} \rangle = \mathrm{Tr}(\mathbf{S_{a,b}} \rho_{a,b}), \tag{8a}$$
$$\langle \mathbf{F_{a,b}} \rangle = \mathrm{Tr}(\mathbf{F_{a,b}} \rho_{a,b}). \tag{8b}$$

Based on these definitions, evolution of the expectation value of the total atomic spin in the two hyperfine states is given by

$$\frac{d\langle \mathbf{F_{a,b}} \rangle}{dt} = \gamma_e (\mathbf{B} + \mathbf{b_e}) \times \langle \mathbf{S_{a,b}} \rangle + \gamma_e (\mathbf{b_n^{AM}} + \mathbf{b_p^{AM}}) \times \langle \mathbf{I_{a,b}} \rangle, \tag{9}$$

where $\gamma_e = \mu_B g_S/\hbar$ denotes the electron gyromagnetic ratio. We can further use the projection theorem[3]

$$\langle \mathbf{S_{a,b}} \rangle = \frac{\langle \mathbf{S_{a,b}} \cdot \mathbf{F_{a,b}} \rangle}{F(F+1)} \langle \mathbf{F_{a,b}} \rangle = \pm \frac{1}{2I+1} \langle \mathbf{F_{a,b}} \rangle, \tag{10}$$

and the relation between total angular momentum $\mathbf{F_{a,b}}$ and the nuclear spin $\mathbf{I_{a,b}}$ and electron spin $\mathbf{S_{a,b}}$

$$\langle \mathbf{I_{a,b}} \rangle = \langle \mathbf{F_{a,b}} \rangle - \langle \mathbf{S_{a,b}} \rangle = \left(1 \mp \frac{1}{2I+1}\right) \langle \mathbf{F_{a,b}} \rangle, \tag{11}$$

to rewrite Eq. (9) into the form

$$\frac{d\langle \mathbf{F_{a,b}} \rangle}{dt} = \pm \frac{\gamma_e}{2I+1} (\mathbf{B} + \mathbf{b_e}) \times \langle \mathbf{F_{a,b}} \rangle + \gamma_e \left(1 \mp \frac{1}{2I+1}\right) (\mathbf{b_n^{AM}} + \mathbf{b_p^{AM}}) \times \langle \mathbf{F_{a,b}} \rangle. \tag{12}$$

In the SERF regime, the expectation values of the projections of the total atomic angular momentum with the magnetic quantum number $m$ are described with spin-temperature distribution[4]

$$\langle F_{a,b},\, m | F_z | F_{a,b},\, m \rangle = \frac{m e^{m\beta}}{Z}, \tag{13}$$

where $\beta$ is the spin-temperature parameter, which can be expressed as a function of the AM polarisation $P$

$$\beta = \ln \left( \frac{1+P}{1-P} \right), \tag{14}$$

and $Z$ is the partition function

$$Z = \sum_{m_a,\, m_b} e^{m\beta}, \tag{15}$$

where indexes $m_a$ and $m_b$ denote the summation over all micro-states of the $F_{a,b}$ hyperfine states.

In the considered case, the hyperfine components of the total atomic angular momentum $\mathbf{F_{a,b}}$ can be expressed through ensemble-averaged total atomic spin $\langle \mathbf{F} \rangle$

$$\langle \mathbf{F_{a,b}} \rangle = k_{a,b} \langle \mathbf{F} \rangle, \tag{16}$$

where $k_{a,b}$ are defined based on the spin-temperature distribution

$$\begin{aligned} k_a &= \frac{\sum_{m_a} m e^{m\beta}}{\sum_{m_a,\, m_b} m e^{m\beta}}, \\ k_b &= \frac{\sum_{m_b} m e^{m\beta}}{\sum_{m_a,\, m_b} m e^{m\beta}}. \end{aligned} \tag{17}$$

After substitution of Eq. (16) into Eq. (12) and adding the equations corresponding to the hyperfine levels, one obtains

$$(k_a+k_b)\frac{d\langle\mathbf{F}\rangle}{dt}=\frac{(k_a-k_b)\gamma_e}{2I+1}(\mathbf{B}+\mathbf{b_e})\times\langle\mathbf{F}\rangle+\gamma_e\left((k_a+k_b)-\frac{k_a-k_b}{2I+1}\right)(\mathbf{b_n^{AM}}+\mathbf{b_p^{AM}})\times\langle\mathbf{F}\rangle, \tag{18}$$

which leads to

$$\frac{d\langle\mathbf{F}\rangle}{dt}=\gamma_e\frac{Q}{2I+1}(\mathbf{B}+\mathbf{b_e})\times\langle\mathbf{F}\rangle+\gamma_e\left(1-\frac{Q}{2I+1}\right)(\mathbf{b_n^{AM}}+\mathbf{b_p^{AM}})\times\langle\mathbf{F}\rangle, \tag{19}$$

where $Q$ denotes a dimensionless gyromagnetic ratio[2]

$$Q=\frac{k_a-k_b}{k_a+k_b}. \tag{20}$$

Using Eq. (13), one can express the dimensionless gyromagnetic ratio Q of AM atoms with a given nuclear spin $I$ using the polarisation[2]

$$Q(P,I=3/2)=2-\frac{4}{3+P^2}, \tag{21a}$$

$$Q(P,I=5/2)=3-\frac{48(1+P^2)}{19+26P^2+3P^4}, \tag{21b}$$

$$Q(P,I=7/2)=\frac{4(1+7P^2+7P^4+P^6)}{11+35P^2+17P^4+P^6}. \tag{21c}$$

In the description, it is convenient to introduce the slowing-down factor of the AM electron-spin expectation value, which takes into account both hyperfine interaction and averaging over hyperfine levels[2]

$$q=\frac{2I+1}{Q}. \tag{22}$$

Applying the projection theorem (10) in the Eq. (19) and taking into account Eq. (22) we can get the evolution of the ensemble-averaged electron spin expectation value **S**, which is directly measured in SERF magnetometers

$$\frac{d\langle\mathbf{S}\rangle}{dt}=\frac{1}{q}\left[\gamma_e(\mathbf{B}+\mathbf{b_e})\times\langle\mathbf{S}\rangle+\gamma_e\left(q-1\right)(\mathbf{b_n^{AM}}+\mathbf{b_p^{AM}})\times\langle\mathbf{S}\rangle\right]. \tag{23}$$

Expressing the electron-spin expectation value $\langle\mathbf{S}\rangle$ with the electron polarisation $\mathbf{P^e}$

$$\mathbf{P^e}=\frac{\langle\mathbf{S}\rangle}{S}, \tag{24}$$

where $S$ is an electron spin quantum number, and introducing the relaxation rate $R^e$ and pumping rate $R_p$ of the electron polarisation, we can write the equation fully describing evolution of the electron polarisation in the SERF magnetometer

$$\frac{d\mathbf{P^e}}{dt}=\frac{1}{q}\left[\gamma_e(\mathbf{B}+\mathbf{b_e})\times\mathbf{P^e}+\gamma_e\left(q-1\right)(\mathbf{b_n^{AM}}+\mathbf{b_p^{AM}})\times\mathbf{P^e}+(\mathbf{s}-\mathbf{P^e})R_p^e-R^e\mathbf{P^e}\right]. \tag{25}$$

This equation is used in the main text for simulation of the SERF-magnetometer response to magnetic and nonmagnetic spin perturbations.

## Co-magnetometer

To describe the coupled spin dynamics of AM and NG atoms subjected to the magnetic and nonmagnetic spin perturbations, we implement a similar approach to that described above. Since we consider the AM-NG co-magnetometer, we can use the result of the previous section to describe the AM electron spin evolution in the presence of magnetic and nonmagnetic fields and only consider the spin dynamics of NG atoms.

The evolution of the NG nuclear spins **K** in the presence of the magnetic field **B** and nonmagnetic perturbation $\mathbf{\Xi}$ is governed by the Hamiltonian $H_{NG}$

$$H_{NG}=-\mu_N g_{NG}\mathbf{B}\cdot\mathbf{K}-\chi_K\mathbf{\Xi}\cdot\mathbf{K}, \tag{26}$$

where $\mu_N$ is the nuclear magneton, $g_{NG}$ is the NG $g$-factor, and $\chi_K$ is the NG nuclear coupling to the anomalous field. In an approach similar to that derived for the AM nuclear anomalous coupling, we divide $\chi_K$ into a part describing the neutron coupling constant $\chi_n$ and proton coupling constant $\chi_p$[1]

$$H_{NG} = -\mu_N g_{NG} \mathbf{B} \cdot \mathbf{K} - (\chi_n \sigma_n^K + \chi_p \sigma_p^K) \boldsymbol{\Xi} \cdot \mathbf{K}, \tag{27}$$

where $\sigma_n^K$ and $\sigma_p^K$ are the neutron and proton fractional contributions to the total NG nuclear spin $K$. It is also convenient to introduce an effective pseudo-magnetic field for the NG spin

$$\mathbf{b_n^{NG}} = \frac{\chi_n \sigma_n^K}{\mu_N g_{NG}} \boldsymbol{\Xi}, \tag{28a}$$

$$\mathbf{b_p^{NG}} = \frac{\chi_p \sigma_p^K}{\mu_N g_{NG}} \boldsymbol{\Xi}. \tag{28b}$$

In such a case, the effective pseudo-magnetic field, affecting the NG nuclear spin $b_N^{NG}$, has the following form

$$\mathbf{b_N^{NG}} = \mathbf{b_n^{NG}} + \mathbf{b_p^{NG}}. \tag{29}$$

The Hamiltonian (27) leads to the following evolution of the NG nuclear spin expectation value

$$\frac{d\langle \mathbf{K} \rangle}{dt} = \gamma_n (\mathbf{B} + \mathbf{b_n^{NG}} + \mathbf{b_p^{NG}}) \times \langle \mathbf{K} \rangle, \tag{30}$$

where $\gamma_n = -\mu_N g_{NG}/\hbar$ denotes nuclear gyromagnetic ratio of the NG.

For the complete description of the AM-NG co-magnetometer, we need to take into account interaction between the AM and NG polarisations. Such interaction manifests as an effective magnetic field originating from polarisation of one atomic ensemble, which affects another ensemble collocated in the same cell[5]

$$\begin{aligned} \frac{d\mathbf{P^e}}{dt} &= \frac{\gamma_e}{q} \mathbf{B^n} \times \mathbf{P^e} = \frac{\gamma_e}{q} \lambda M^n \mathbf{P^n} \times \mathbf{P^e}, \\ \frac{d\mathbf{P^n}}{dt} &= \gamma_n \mathbf{B^e} \times \mathbf{P^n} = \gamma_n \lambda M^e \mathbf{P^e} \times \mathbf{P^n}, \end{aligned} \tag{31}$$

where $\mathbf{B^e}$ and $\mathbf{B^n}$ stand for the fields stemming from the AM and NG polarisations, respectively. Those fields are defined by the coupling parameter[5] $\lambda$ and the maximal magnetisations of the AM and NG ($M^e$ and $M^n$, respectively). The NG nuclear polarisation $\mathbf{P^n}$ is defined in a following way (here $K$ is NG nuclear spin quantum number)

$$\mathbf{P^n} = \frac{\langle \mathbf{K} \rangle}{K}. \tag{32}$$

Rewriting Eq. (30) in terms of the polarisation and adding the interaction between the polarisations from Eq. (31) along with the collisional relaxation of AM $R_c^e$ and NG $R_c^n$, we get the complete description of the co-magnetometer in external fields by combining this result with Eq. (25)

$$\begin{cases} \frac{d\mathbf{P^e}}{dt} &= \frac{1}{q} \left[ \gamma_e (\mathbf{B} + \mathbf{b_e} + \lambda M^n \mathbf{P^n}) \times \mathbf{P^e} + (q-1)\gamma_e \mathbf{b_N^{AM}} \times \mathbf{P^e} + R_{se}^{ne} \mathbf{P^n} + (\mathbf{s} - \mathbf{P^e}) R_p - (R_c^e + R_{se}^{en}) \mathbf{P^e} \right], \\ \frac{d\mathbf{P^n}}{dt} &= \gamma_n (\mathbf{B} + \mathbf{b_N^{NG}} + \lambda M^e \mathbf{P^e}) \times \mathbf{P^n} + R_{se}^{en} \mathbf{P^e} - (R_{se}^{ne} + R_c^n) \mathbf{P^n}, \end{cases} \tag{33}$$

where $R_{se}^{ne}$ and $R_{se}^{en}$ rates of spin-exchange transfer of the polarisation from NG to AM and from AM to NG, respectively. This set of equations is used in our main manuscript to analyse the response of the co-magnetometer to magnetic and pseudo-magnetic spin perturbations.

## Simulation parameters

For the numerical simulations presented in the main text, the parameters, given in Table 1, were used. The maximum effective fields associated with interaction between the AM and NG polarisations were calculated based on

$$\begin{aligned} \lambda M^e &= \frac{8\pi\kappa}{3} n_{AM} \mu_{AM} S, \\ \lambda M^n &= \frac{8\pi\kappa}{3} N_{NG} \mu_{NG} K, \end{aligned} \tag{34}$$

where $\kappa$ is the enhancement factor[5] (see Table 1), $n_{AM}$ and $n_{NG}$ are concentrations of the AM and NG atoms, $\mu_{AM}$ and $\mu_{NG}$ are the magnetic moments of the AM atoms and NG nuclei. We estimate the concentration of $^{39}K$ at 190°C using the phenomenological formula based on Ref.[6]

$$n_K = \frac{1}{k_B T} 10^{3.4077-4453/T} \text{ cm}^{-3}. \tag{35}$$

**Table 1.** Complete list of parameters used for the simulations.

| Parameter | Value | Units |
|---|---|---|
| Electron gyromagnetic ratio $\gamma_e$ | $2\pi \cdot 2802.5$ | $\text{s}^{-1}\text{mG}^{-1}$ |
| AM pumping rate $R_p$ | 600 | $\text{s}^{-1}$ |
| AM total relaxation rate $R^e$ | 1200 | $\text{s}^{-1}$ |
| AM steady state polarisation $P_0^e$ | 0.5 | |
| AM nuclear spin $I$ | 3/2 | $\hbar$ |
| System temperature | 190 | °C |
| AM concentration | $10^{14}$ | $\text{cm}^{-3}$ |
| AM-NG enhancement factor $\kappa$ | 6 | |
| NG concentration | 3.5 | 1 amg $\approx 2.69 \cdot 10^{19}$ $\text{cm}^{-3}$ |
| NG gyromagnetic ratio $\gamma_n$ | $2\pi \cdot 3.24$ | $\text{s}^{-1}\text{mG}^{-1}$ |
| NG relaxation rate $R^n$ | $5 \cdot 10^{-5}$ | $\text{s}^{-1}$ |
| NG steady state polarisation $P_0^n$ | 0.05 | |
| NG nuclear spin $K$ | 1/2 | $\hbar$ |
| Maximum AM electron effective field $\lambda M^e$ | 0.067 | mG |
| Maximum NG effective field $\lambda M^b$ | 25 | mG |
| Compensation magnetic field $B_c$ | -1.31 | mG |
| Amplitude of the probe periodic perturbation $A_0$ (normalised to NG proton perturbation) | $10^{-4}$ | mG |

## Analytical solution for the frequency response of the co-magnetometer

In this section, we present a simplified model that enables derivation of analytical relations for the response of the co-magnetometer to the magnetic and nonmagnetic perturbations. The model follows the investigations presented in Ref.[7] and extends the previous results by analysing the response to non-magnetic spin couplings. The obtained results are used to validate the numerical simulations presented in the main text.

As the starting point, we take the system described by Eqs. 11 from the main text. For the analysis, we introduce a complex notation of the polarisation and transverse fields

$$\begin{aligned} P_\perp^{e,n} &= P_x^{e,n} + iP_y^{e,n}, \\ B_\perp &= B_x + iB_y. \end{aligned} \tag{36}$$

The effective pseudo-magnetic fields due to nonmagnetic couplings can be introduced in similar manner. We also assume that the transverse perturbations are weak, so that, during the system evolution the changes of the longitudinal polarisations $P_z^e$ and $P_z^n$ are negligible

$$P_z^{e,n} \approx P_0^{e,n}.$$

Under these assumptions and notation, the evolution of the transverse polarisation $\mathbf{P}_\perp = (P_\perp^e, P_\perp^n)^T$ can be presented as

$$\frac{d}{dt}\mathbf{P}_\perp = \begin{pmatrix} i\frac{\gamma_e}{q}(B_z + \lambda M^n P_z^n) - \frac{R^e}{q} & -i\frac{\gamma_e}{q}\lambda M^n P_z^e \\ -i\gamma^n \lambda M^e P_z^n & i\gamma^n(B_z + \lambda M^e P_z^e) - R^n \end{pmatrix} \mathbf{P}_\perp - i \begin{pmatrix} \frac{\gamma_e}{q} P_z^e \left[B_\perp + b_\perp^e + (q-1) b_{N\perp}^{AM}\right] \\ \gamma^n P_z^n (B_\perp + b_{N\perp}^{NG}) \end{pmatrix}. \tag{37}$$

At the compensation point defined in Eq. (**??**) the above equation can be written as

$$\frac{d}{dt}\mathbf{P}_\perp = -\begin{pmatrix} i\frac{\gamma_e}{q}\lambda M^e P_z^e + \frac{R^e}{q} & i\frac{\gamma_e}{q}\lambda M^n P_z^e \\ i\gamma^n \lambda M^e P_z^n & i\gamma^n \lambda M^n P_z^n + R^n \end{pmatrix} \mathbf{P}_\perp - i \begin{pmatrix} \frac{\gamma_e}{q} P_z^e \left[B_\perp + b_{e\perp} + (q-1) b_{N\perp}^{AM}\right] \\ \gamma^n P_z^n (B_\perp + b_{N\perp}^{NG}) \end{pmatrix}. \tag{38}$$

We solve Eq. (38) for each type of perturbation independently.

In the complex notation magnetic or nonmagnetic perturbations defined in Eq. (**??**) have the following form

$$A_\perp = iA_0 \sin(2\pi\nu t) = \frac{A_0}{2}(e^{i2\pi\nu t} - e^{-i2\pi\nu t}), \tag{39}$$

where $\nu$ is the perturbation frequency.

As we are interested in a dynamic steady-state polarisation, the polarisation $\mathbf{P}_\perp$ needs to take the form

$$\mathbf{P}_\perp = \mathbf{P}_+ e^{2\pi\nu t} + \mathbf{P}_- e^{-2\pi\nu t}, \tag{40}$$

where $\mathbf{P}_\pm$ are the amplitudes of the components rotating clockwise and counter-clockwise with the frequency $\nu$. The amplitudes $\mathbf{P}_\pm$ can be calculated using Eq. (38) and taking into account the orthogonality of $e^{\pm 2\pi\nu t}$.

Since the experiment is sensitive to the AM polarisation, in our analysis, we only focus on the polarisation. Below, we present the solutions of Eq. (38) for magnetic, electron nonmagnetic, and nuclear nonmagnetic perturbations, denoted with the subscripts *magn*, *el*, and *nucl*, respectively

$$P^e_{\pm,\,magn} = -\frac{\gamma^e P^e_z}{2q} \frac{(2\pi\nu \mp iR^n)B_0}{[2\pi\nu \pm (\omega^e - iR^e/q)][2\pi\nu \pm (\omega^n - iR^n)] - \omega^e\omega^n}, \tag{41a}$$

$$P^e_{\pm,\,el} = -\frac{\gamma^e P^e_z}{2q} \frac{(2\pi\nu \pm \omega^n \mp iR^n)A^e_0}{[2\pi\nu \pm (\omega^e - iR^e/q)][2\pi\nu \pm (\omega^n - iR^n)] - \omega^e\omega^n}, \tag{41b}$$

$$P^e_{\pm,\,nucl} = -\frac{\gamma^e P^e_z}{2q} \frac{(2\pi\nu \pm \omega^n \mp iR^n)(q-1)A^{AM}_{0N} \mp \omega^n A^{NG}_{0N}}{[2\pi\nu \pm (\omega^e - iR^e/q)][2\pi\nu \pm (\omega^n - iR^n)] - \omega^e\omega^n}, \tag{41c}$$

where $B_0$ is the amplitude of the periodic magnetic perturbation [compare to $A_0$ in Eq. (39)], $A^e_0$ is the amplitude of the electron nonmagnetic perturbation, $A^{AM}_{0N}$ and $A^{NG}_{0N}$ are the amplitudes of the nuclear nonmagnetic perturbation of AM and NG, respectively, with all $A$'s given in effective pseudo-magnetic units. The AM and NG Larmor angular frequencies $\omega^e$ and $\omega^n$ are given by

$$\begin{aligned} \omega^e &= \frac{\gamma^e}{q}\lambda M^e P^e_z, \\ \omega^n &= \gamma^n \lambda M^n P^n_z. \end{aligned} \tag{42}$$

Finally, the $x$-component of the AM polarisation, i.e., the optically detectable component of the electron polarisation, can be calculated as

$$P^e_x = \mathrm{Re}(\mathbf{P}^\mathbf{e}_\perp) = \left[\mathrm{Re}(P^e_+) + \mathrm{Re}(P^e_-)\right]\cos(2\pi\nu t) + \left[\mathrm{Im}(P^e_-) - \mathrm{Im}(P^e_+)\right]\sin(2\pi\nu t) = P^e_c \cos(2\pi\nu t) + P^e_s \sin(2\pi\nu t). \tag{43}$$

To compare this solution with the results of numerical simulations, one should present the former in the following form

$$P^e_x = S' \sin(2\pi f' t + \phi'), \tag{44}$$

where the parameters $S'$, $f'$, and $\phi'$ are related to the system parameter by

$$\begin{aligned} f' &= \nu, \\ S' &= \sqrt{(P^e_s)^2 + (P^e_c)^2}, \\ \phi' &= \arccos\left(\frac{P_s}{S'}\right) \mathrm{sgn}\left[\arcsin\left(\frac{P_c}{S'}\right)\right], \end{aligned} \tag{45}$$

with sgn being the function returning the sign of the argument. It should be noted that the solution for the SERF magnetometer can be obtained with the same method, by assuming $P^n_z = P^n_\perp = 0$.

As shown in Fig. 1 in the main text, the analytical solution agrees well with numerical simulations (difference at a level of 0.2% or smaller), for both the SERF magnetometer and co-magnetometer, which validates the developed analytical model.

## References


**1.** Jackson Kimball, D. F. Nuclear spin content and constraints on exotic spin-dependent couplings. *New J. Phys.* **17**, 073008, DOI: 10.1088/1367-2630/17/7/073008 (2015).

**2.** Savukov, I. M. & Romalis, M. V. Effects of spin-exchange collisions in a high-density alkali-metal vapor in low magnetic fields. *Phys. Rev. A* **71**, 023405, DOI: 10.1103/PhysRevA.71.023405 (2005).

**3.** Steck, D. A. Quantum and atom optics. available online at http://steck.us/teaching (revision 0.13.4) (2020).

**4.** Budker, D. & Jackson Kimball, D. (eds.) *Optical Magnetometry* (Cambridge University Press, 2013).

**5.** Kornack, T. W. & Romalis, M. V. Dynamics of two overlapping spin ensembles interacting by spin exchange. *Phys. Rev. Lett.* **89**, 253002, DOI: 10.1103/PhysRevLett.89.253002 (2002).

**6.** Alcock, C. B., Itkin, V. P. & Horrigan, M. K. Vapour pressure equations for the metallic elements: 298–2500 K. *Can. Metall. Q.* **23**, 309–313 (1984).

**7.** Lee, J. *New constraints on the axion's coupling to nucleons from a spin-mass interaction limiting experiment (SMILE)*. Ph.D. thesis, Princeton University (2019).
